# A dataset for the perceived vulnerability to disease scale in Japan before the spread of COVID-19

**DOI:** 10.12688/f1000research.23713.2

**Published:** 2020-07-22

**Authors:** Yuki Yamada, Haoqin Xu, Kyoshiro Sasaki

**Affiliations:** 1 Faculty of Arts and Science, Kyushu University, Fukuoka, Japan; 2 Graduate School of Human-Environment Studies, Kyushu University, Fukuoka, Japan; 3 Faculty of Informatics, Kansai University, Takatsuki, Japan

**Keywords:** coronavirus, disgust, emotion, Japanese, perceived infectability, germ aversion

## Abstract

The COVID-19 outbreak is a worldwide medical and epidemiological catastrophe, and the number of psychological studies concerning COVID-19 is growing daily. Such studies need baseline data from before the COVID-19 outbreak for comparison, but such datasets have not yet been accumulated and shared. Here, we provide a dataset on the perceived vulnerability to disease scale for 1382 Japanese participants obtained through an online survey conducted in 2018 that will be useful for comparison with current or post-COVID-19 perceived vulnerability to disease data.

## Introduction

Currently, a new type of coronavirus infection (coronavirus disease 2019; COVID-19) is spreading on a global scale. Although the details of this infection are not yet clear, it is rapidly spreading in many countries, and the strength of the infection is likely to be great. In response to this unprecedented situation, governments are asking people to take social distancing measures and limit their outside activities. Under such threats and political measures, the state of mind of the people must also be quite different from before. Therefore, many social and behavioral studies of such factors including people’s political attitudes, controllability, emotional states, anxiety, and stress in COVID-19 situations are being conducted simultaneously and rapidly ( Van Bavel *et al.,* 2020). However, it is difficult to predict how long the current pandemic will last, and even if the situation is under control, it is unclear whether the psychological traits of people in the post-COVID-19 world who experienced this pandemic will be similar to those before COVID-19. Thus, such survey studies need pre-outbreak data as a baseline for comparison and data obtained before the COVID-19 outbreak are of great importance. Accordingly, we here provide a dataset for the perceived vulnerability to disease scale obtained in Japan in 2018 ( Yamada, 2020).

**Figure 1.**
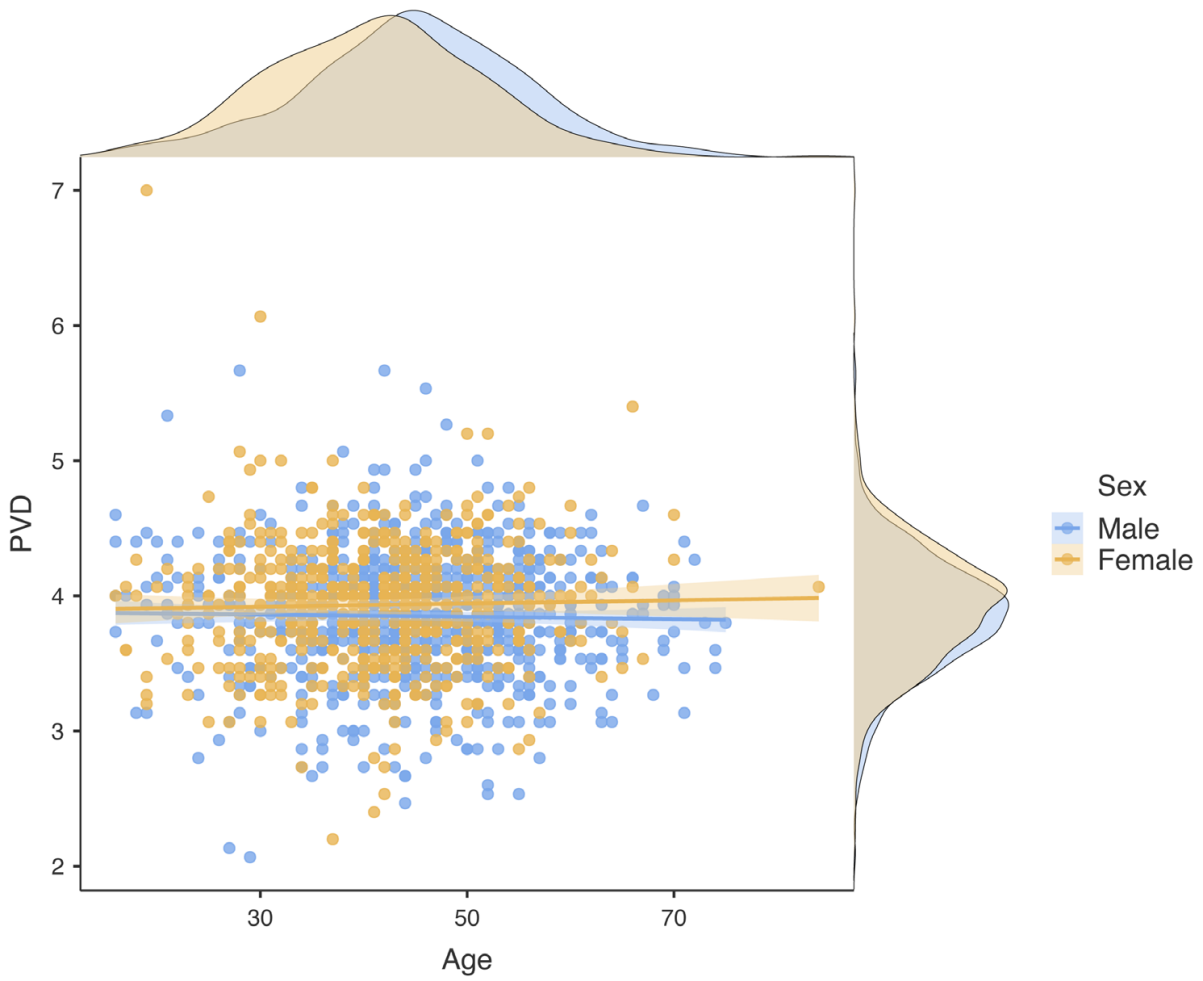
The relation between the individual PVD scale score and age for male and female participants. We excluded the data of the participants whose sex was unknown from this figure because their sample size was only 16.

 With the spread of COVID-19 and under the guidance of the World Health Organization (WHO), people have begun to wash their hands more often. Concomitantly, we have become more afraid of infection than ever before. People have begun to disinfect various places and objects with alcohol and to wear masks. These behaviors are based on a heightened perceived vulnerability to disease (PVD). A psychological scale has been developed to measure this tendency ( Duncan *et al.,* 2009). The PVD scale is composed of two subscales: “Perceived Infectability,” which is related to the beliefs of one’s own susceptibility to infecting diseases, and “Germ Aversion,” which is related to an awareness of discomfort in situations with a high likelihood of infection with a pathogen. This scale has already been localized in Japan ( Fukukawa *et al.,* 2014). It has also been translated not only in Japan but other countries as well ( Ahmadzadeh *et al.,* 2013; Klavina *et al.,* 2011; Prokop *et al.,* 2010; Skolnick & Dzokoto, 2013). In the early stages of the COVID-19 spread, the PVD was used to confirm concurrent validity of the scale regarding the COVID-19 ( Ahorsu *et al.*, 2020). Moreover, a Chinese study had already compared PVD scale scores with those of other countries ( Goh, 2020); this was a cross-sectional study and there was no baseline. These results suggest that the generality of this scale and the necessity of baseline data are both striking. Therefore, we provide data on the pre-pandemic PVD scale for use in comparative studies ( Yamada, 2020). The distribution of the individual PVD scale score is shown in Figure 1.

## Methods

### Participants

We recruited a maximum of 2000 participants online through Yahoo! Crowdsourcing Service and recorded the data collected during the survey period. As a result, a total of 1428 Japanese people in Japan participated in this survey (868 men, 543 women, 17 unknown; mean age ± SD = 43.40 ± 10.62 years).

### Scale

We used the Japanese version of the PVD scale developed by Fukukawa *et al.* (2014). The scale consists of a total of 15 items. Each item was scored on a seven-point scale (1: *strongly disagree*, 7: *strongly agree*). Items 3, 5, 11, 12, 13, and 14 were reverse-scaled items. All the items of this scale are available from the original papers (English: Duncan *et al.,* 2009; Japanese: Fukukawa *et al.,* 2014).

### Procedure

The survey was conducted from September 22–23, 2018. Participants accessed the Yahoo! Crowdsourcing service page for the link to the web address of the survey page on Google Forms. The participants were first asked to input their age and sex (male, female, or other). The order of items on the scale was randomized across participants based on the setting of Google Forms. In order to check whether the participants were concentrating on the task, a calculation problem (171 − 169 = ?) was inserted after the 7th item of the PVD scale as an attention check question to identify respondents who do not answer seriously ( Sasaki & Yamada, 2019). After the survey, the participants received 10 T-points (Japanese point service, in which one T-point is worth one JPY) as a reward.

### Inclusion

The survey was posted on the website of the crowdsourcing service and users of the service were free to view it and participate in it.

### Exclusion

We excluded participants who gave an incorrect answer to the attention check question. As a result, we eliminated the data of 46 participants. We present the remaining dataset for 1382 participants (833 men, 533 women, 16 unknown; mean age ± SD = 43.46 ± 10.62 years) as a relatively reliable one.

### Ethical approval and consent to participate

The present study received approval from the psychological research ethics committee of the Faculty of Human-Environment Studies at Kyushu University (approval number: 2016-017). Completion of the survey was taken as consent to participate from participants. Participants had the right to withdraw from the survey at any time without providing a reason. Although we did not obtain personal information about the participants, as this was a crowdsourced survey, it was explained to them that their responses would not be tied to them personally.

## Data availability

### Underlying data

Open Science Framework: Japan PVD 2018. https://doi.org/10.17605/OSF.IO/QW2AF; registration DOI https://doi.org/10.17605/OSF.IO/7Y4AV ( Yamada, 2020).

This project contains the following underlying data:

- PVDJapan2018.xlsx. (The dataset.)
- Description of Dataset.txt.

Data are available under the terms of the Creative Commons Zero “No rights reserved” data waiver (CC0 1.0 Public domain dedication).
