# Supplementary material for: A dataset for the perceived vulnerability to disease scale in Japan before the spread of COVID-19
Source: F1000Res. 2020 Jul 22;9:334. Originally published 2020 May 6. [Version 2] doi: 10.12688/f1000research.23713.2 (PMC7468562; doi:10.12688/f1000research.23713.2)
Supplement: Peer review history

# Peer review: Reviewer response for version 2

**DOI:** 10.5256/f1000research.28113.r67735

**Published:** 2020-09-02

**Authors:** Amir H. Pakpour

**Affiliations:** 1 Research Institute for Prevention of Non-Communicable Diseases, Qazvin University of Medical Sciences, Qazvin, Iran

The authors have addressed my previous concerns. It is acceptable

Are sufficient details of methods and materials provided to allow replication by others?

No

Is the rationale for creating the dataset(s) clearly described?

Yes

Are the datasets clearly presented in a useable and accessible format?

Yes

Are the protocols appropriate and is the work technically sound?

Partly

Reviewer Expertise:

Psychology, methodology, Biostatistics

I confirm that I have read this submission and believe that I have an appropriate level of expertise to confirm that it is of an acceptable scientific standard.

# Peer review: Reviewer response for version 1

**DOI:** 10.5256/f1000research.26163.r64012

**Published:** 2020-07-14

**Authors:** Amir H. Pakpour

**Affiliations:** 1 Research Institute for Prevention of Non-Communicable Diseases, Qazvin University of Medical Sciences, Qazvin, Iran

Dear Author(s),

 Please 

1. The aim of this study is vague. The abstract is incomplete and the results and the method parts are not accessible. In addition to this, your keywords are not able to reflect the whole details of the study.
2. One of the psychological scales was introduced, however, there is not any hint to find out about that.
3. In the method part, there is not solid ground to opt Web-sampling? it could lead to bias definitely.

1. Is there any previous knowledge to select age and sex as covariates?
2. I would suggest the authors add the following citation: 
  Ahorsu, D. K., Lin, C. Y., Imani, V., Saffari, M., Griffiths, M. D., & Pakpour, A. H. (2020). The fear of COVID-19 scale: development and initial validation. *International journal of mental health and addiction*. ^1^

Are sufficient details of methods and materials provided to allow replication by others?

No

Is the rationale for creating the dataset(s) clearly described?

Yes

Are the datasets clearly presented in a useable and accessible format?

Yes

Are the protocols appropriate and is the work technically sound?

Partly

Reviewer Expertise:

Psychology, methodology, Biostatistics

I confirm that I have read this submission and believe that I have an appropriate level of expertise to confirm that it is of an acceptable scientific standard, however I have significant reservations, as outlined above.

## References

[1] : The Fear of COVID-19 Scale: Development and Initial Validation. *Int J Ment Health Addict*.2020; 10.1007/s11469-020-00270-8 1-9 10.1007/s11469-020-00270-8 32226353PMC7100496

# Response

**Published:** 2020-07-15

**Authors:** Yuki Yamada

**Affiliations:** Kyushu University, Japan

Thank you for reviewing our data paper. We were happy to address each comment.

*1. The aim of this study is vague. The abstract is incomplete and the results and the method parts are not accessible. In addition to this, your keywords are not able to reflect the whole details of the study.*

**Reply:** Thank you for your comment. Our manuscript was submitted as *Data Notes*, in which, the paper aims to provide a valuable dataset and promote its reuse. Further, this point was already apparent in the first draft. Generally, *Data Notes* should explain how the data was created without including any analyses or conclusions. As well, we followed the article guidelines, which are available at https://f1000research.com/for-authors/article-guidelines. Therefore, based on the guidelines, we believe that our abstract was not incomplete.

*2. One of the psychological scales was introduced, however, there is not any hint to find out about that.*

**Reply:** The present study used only the Japanese version of the perceived vulnerability to disease (PVD) scale (Fukukawa et al., 2014); we assume that you are referring to this scale. This scale is a just localized version of the original PVD scale (Duncan et al., 2009); the contents in the Japanese version of the PVD scale are also identical to the original scale. Furthermore, we already clarified this point in the Introduction of the first draft.

*3. n the method part, there is not solid ground to opt Web-sampling? it could lead to bias definitely.*

**Reply:** Thank you very much for your remark. Although it is unclear to us what kind of bias you were referring to regarding the web sampling, we believe that those who will use this dataset will carefully address any areas of concern. To that end, we explicitly stated that this data was obtained by web-sampling and provided more detailed data characteristics, as responded in 2-4.

*4. Is there any previous knowledge to select age and sex as covariates?*

**Reply:** As per our response in 2-1, *Data Notes* do not have to include any analyses or conclusions. Moreover, age and sex as covariates are dependent on the purpose of using this dataset and should not be predicated here; therefore, those using the dataset should address age and sex as covariates as they deem necessary. But just for information, we would show a graph representing the relation between the individual PVD scale score and age for each gender for future data users.

*5. I would suggest the authors add the following citation: Ahorsu, D. K., Lin, C. Y., Imani, V., Saffari, M., Griffiths, M. D., & Pakpour, A. H. (2020). The fear of COVID-19 scale: development and initial validation. International journal of mental health and addiction. ^1^*

**Reply:** Thank you for this valuable information. We have cited this article in the revised manuscript.

 We express our sincere gratitude for your time and effort for the valuable comments and suggestions, and hope that our revised manuscript is now suitable for approval.

# Peer review: Reviewer response for version 1

**DOI:** 10.5256/f1000research.26163.r63128

**Published:** 2020-05-26

**Authors:** Shu Imaizumi

**Affiliations:** 1 Institute for Education and Human Development, Ochanomizu University, Tokyo, Japan

The authors provide a dataset of the PVD scale from a Japanese sample in September 2018, which would serve as a baseline for comparison with the PVD in the current and post COVID-19 situations. The study will give not only a dataset but also an important message for future studies on the changes in human behavior during and after this COVID-19 period. I would like to give some minor comments.

**Introduction:**

- To be precise, COVID-19 is an abbreviation for coronavirus disease 2019.
- “perceived infectability” and “germ aversion” may be capitalized, according to Duncan *et al.* (2009).
- It might be better to clarify that Goh (2020) was a cross-sectional study (i.e., no comparison with pre-COVID-19 data) to underscore the necessity of baseline.

**Participants, Exclusion:**

- It would be better to report SD of participants’ age.

 

**Procedure:**

- Please clarify whether the calculation problem was inserted between or after the PVD scale items.

Are sufficient details of methods and materials provided to allow replication by others?

Yes

Is the rationale for creating the dataset(s) clearly described?

Yes

Are the datasets clearly presented in a useable and accessible format?

Yes

Are the protocols appropriate and is the work technically sound?

Yes

Reviewer Expertise:

Cognitive psychology

I confirm that I have read this submission and believe that I have an appropriate level of expertise to confirm that it is of an acceptable scientific standard.

# Response

**Published:** 2020-07-15

**Authors:** Yuki Yamada

**Affiliations:** Kyushu University, Japan

*The authors provide a dataset of the PVD scale from a Japanese sample in September 2018, which would serve as a baseline for comparison with the PVD in the current and post COVID-19 situations. The study will give not only a dataset but also an important message for future studies on the changes in human behavior during and after this COVID-19 period. I would like to give some minor comments.*

**Reply:** Thank you very much for your positive and valuable comments. We want to address your remarks with sincerity.

*To be precise, COVID-19 is an abbreviation for coronavirus disease 2019.*

*“perceived infectability” and “germ aversion” may be capitalized, according to Duncan et al. (2009).*

**Reply:** Following your suggestions, we have revised the manuscript accordingly.

*It might be better to clarify that Goh (2020) was a cross-sectional study (i.e., no comparison with pre-COVID-19 data) to underscore the necessity of baseline.*

**Reply:** Thank you for this valuable suggestion. We have clarified that Goh (2020) was a cross-sectional study in the revised manuscript.

*It would be better to report SD of participants’ age.*

**Reply: **We have calculated the *SD* (= 10.62) of participants’ age and indicated this in the revised manuscript.

                            

*Please clarify whether the calculation problem was inserted between or after the PVD scale items.*

**Reply: **Based on this comment, we have clarified that the calculation problem was inserted after the 7th item of the PVD scale.
